# Assessing visual search performance using a novel dynamic naturalistic scene

**DOI:** 10.1167/jov.21.1.5

**Published:** 2021-01-11

**Authors:** Christopher R. Bennett, Peter J. Bex, Lotfi B. Merabet

**Affiliations:** 1 The Laboratory for Visual Neuroplasticity, Department of Ophthalmology, Massachusetts Eye and Ear, Harvard Medical School, Boston, MA, USA; 2 Translational Vision Lab, Department of Psychology, Northeastern University, Boston, MA, USA

**Keywords:** virtual reality, visual search, eye tracking, motion processing, dynamic scenes, saccades, pursuit

## Abstract

Daily activities require the constant searching and tracking of visual targets in dynamic and complex scenes. Classic work assessing visual search performance has been dominated by the use of simple geometric shapes, patterns, and static backgrounds. Recently, there has been a shift toward investigating visual search in more naturalistic dynamic scenes using virtual reality (VR)-based paradigms. In this direction, we have developed a first-person perspective VR environment combined with eye tracking for the capture of a variety of objective measures. Participants were instructed to search for a preselected human target walking in a crowded hallway setting. Performance was quantified based on saccade and smooth pursuit ocular motor behavior. To assess the effect of task difficulty, we manipulated factors of the visual scene, including crowd density (i.e., number of surrounding distractors) and the presence of environmental clutter. In general, results showed a pattern of worsening performance with increasing crowd density. In contrast, the presence of visual clutter had no effect. These results demonstrate how visual search performance can be investigated using VR-based naturalistic dynamic scenes and with high behavioral relevance. This engaging platform may also have utility in assessing visual search in a variety of clinical populations of interest.

## Introduction

Visual search can be very demanding in our dynamic and ever-changing surroundings. For example, the task of finding and following a person in a crowd requires that a specific target be identified and continuously tracked within a complex moving scene. Much work has explored various aspects of visual search based on a variety of behavioral testing paradigms (Rao, Zelinsky, Hayhoe, & Ballard, 2002; Treisman, 1988; Wolfe, 2007; Zelinsky, Zhang, Yu, Chen, & Samaras, 2006; for review, see Eckstein, 2011; Wolfe & Horowitz, 2017), and the underlying neural correlates associated with various search tasks have also been explored (Bundesen, Habekost, & Kyllingsbæk, 2005; Desimone & Duncan, 1995; Eimer, 2014; Luck & Hillyard, 1994).

Visual search performance has been investigated in the context of manipulating task demands and parameters and serves as an important paradigm for exploring serial and parallel deployment of attention (Treisman & Gelade, 1980). For example, increasing the number of surrounding elements has little or no effect on search times when the target differs from distractors by a single feature such as shape, size, or color (Nakayama & Silverman, 1986; Teichner & Krebs, 1974). In contrast, search time for conjunctions of two or more features increases with the number of distractors (Palmer, 1994; Treisman, 1988; Wolfe, 1994), but not in all cases (Nakayama & Silverman, 1986). Other non-target-related stimulus features can also affect the visual search process by impacting strategy usage and overall efficiency (Horowitz, Wolfe, DiMase, & Klieger, 2007; Moran, Zehetleitner, Liesefeld, Müller, & Usher, 2016). For example, the presence of environmental clutter and altering the trajectory of the target being followed can also make search more difficult (Bravo & Farid, 2004; Matsuno & Tomonaga, 2006; Rosenholtz, Li, & Nakano, 2010).

Understanding how these manipulations affect search performance is useful in characterizing the perceptual limits of the human visual system. Classic work has largely focused on simple static two-dimensional geometric targets, patterns, and backgrounds; however, the nature of these visual stimuli differs considerably from everyday experience. A number of groups have examined visual search using more naturalistic images (e.g., Ehinger, Hidalgo-Sotelo, Torralba, & Oliva, 2009; Hwang, Wang, & Pomplun, 2011; Torralba, Oliva, Castelhano, & Henderson, 2006; Võ & Wolfe, 2012), but these studies are limited to the presentation of static images. Other studies have explored search performance using real-world environments and three-dimensional (3D) naturalistic environments (Foulsham, Chapman, Nasiopoulos, & Kingstone, 2014; Kuliga, Thrash, Dalton, & Hölscher, 2015; Li, Aivar, Kit, Tong, & Hayhoe, 2016). Still, maintaining control over stimulus parameters in real-world studies can be difficult, and testing has often been limited to viewing static scenes. Despite these efforts, there still remains a need to develop adaptable and controllable stimuli for the purposes of investigating visual search performance using behavioral paradigms that can be considered as more ecologically valid (Bennett, Bex, Bauer, & Merabet, 2019; Helbing, Draschkow, & Võ, 2020; Parsons, 2011; Parsons, 2015; Parsons & Duffield, 2019; for further discussion, see Holleman, Hooge, Kemner, & Hessels, 2020). In this direction, virtual reality (VR) has gained considerable interest as a way to approach issues related to task realism, immersion, adaptability, and experimental control, and it has even found a growing application in clinical and behavioral neuroscience research (for reviews, see Bouchard, 2019; Parsons & Phillips, 2016; Tarr & Warren, 2002). There are numerous benefits to using VR as a tool for performance assessment and training, including task flexibility, experimental control and safety, objective data capture, and high participant engagement and motivation, as well as the ability to mimic real-world scenarios with a high level of behavioral relevance (Dickey, 2003; Loomis, Blascovich, & Beall, 1999; Merchant, Goetz, Cifuentes, Keeney-Kennicutt, & Davis, 2014; Parsons & Phillips, 2016). Further, the combination of VR with informative measures of performance affords a high degree of experimental control, allowing the effects of manipulating task difficulty and other environmental factors to be examined (Bennett, Corey, Giudice, & Giudice, 2016; Loomis et al., 1999; Zyda, 2005).

From a clinical perspective, VR-based assessments may also be very helpful in characterizing higher order perceptual abilities and deficits that cannot be characterized using standard ophthalmic assessments such as visual acuity and visual field perimetry (Bennett et al., 2019; Colenbrander, 2005). This is particularly relevant with respect to searching and tracking targets in a dynamic and complex scene. In contrast to static images, searching for moving targets in dynamic scenes requires that the spatial locations of various elements be continuously integrated and updated (Awh, Barton, & Vogel, 2007; Gallwey & Drury, 1986; Müller & Rabbitt, 1989; Tsotsos, 1989; Wolfe & Horowitz, 2017). Previous studies have attempted to investigate these issues employing a variety of visual stimuli, such as biological form from motion, optic flow fields (including random dot kinematograms), and multiple object tracking (Johansson, 1973; Newsome & Paré, 1988; Pylyshyn & Storm, 1988). Although these stimuli offer a high degree of stimulus control, they nonetheless remain limited in terms of their ecological validity and behavioral relevance. In other words, it is often difficult to determine how behavioral results obtained from these stimuli translate to visual processing abilities in real-world tasks and situations.

To address this limitation, we developed a dynamic visual search task using a desktop VR-based simulation combined with eye tracking. In this task, referred to as the “virtual hallway,” participants were required to search, locate, and pursue a specific target individual (i.e., the principal of a fictitious school) walking in a crowded hallway. As part of the stimulus design, we incorporated a means to modify task demands by varying crowd density and the presence of visual clutter in the environment. Using this testing paradigm, we proposed the following hypotheses and potential observations: (a) visual search performance (as indexed by a variety of eye-tracking metrics) would show greater impairment with increasing crowd density (i.e., surrounding distractors), and (b) the presence of visual clutter would also impair search performance. Early results using this testing paradigm have been published (Bennett, Bailin, Gottlieb, Bauer, Bex, & Merabet, 2018a).

## Methods

### Participants

Thirty-five individuals (25 female, 10 male) with neurotypical development and who were between the ages of 14 and 28 years (mean ± *SD*, 21.2 ± 4.4 years) participated in the study. All participants had normal or corrected-to-normal visual acuity and no previous history of ophthalmic (e.g., strabismus, amblyopia) or neurodevelopmental (e.g., epilepsy, attention deficit disorder) conditions. The study was approved by the institutional review board of Massachusetts Eye and Ear, and written informed consent was obtained from all the participants prior to commencing the study.

### Initial focus group work and motivation for study

Our group develops novel assessments designed to evaluate higher order processing abilities and deficits that cannot be characterized using standard ophthalmic measures of visual function. Prior to commencing the study, we carried out a focus group study comprised of potential clinical participants, parents, teachers of the visually impaired, and clinicians to identify real-world scenarios that were deemed particularly challenging. Through a series of questionnaires (quantified using Likert scales and open-ended questions) and iterative testing, we concluded that identifying and following a person walking in a crowd was particularly difficult for individuals. Furthermore, the size of the crowd and the presence of visual clutter were also identified as factors of interest that could potentially influence performance.

Overall scores from respondents regarding visual stimulus factors such as realism and feature importance were interpreted as supporting the high behavioral relevance of our visual task design (see details regarding visual stimulus design below). Here, we present results from a sample of individuals with neurotypical development in order to characterize baseline performance on the dynamic visual search task we designed based on this focus group work.

### Visual stimulus design and manipulations

To assess performance searching for an individual walking in a crowd, we developed a VR-based simulation referred to as the “virtual hallway.” The simulation was a rendering of a hallway of a typical school with a crowd of people walking around the observer. The scene was presented in a dynamic, continuous fashion and viewed from a fixed, first-person perspective. Participants were instructed to search, locate, and pursue a specific target individual (the principal of the fictitious school) walking in a crowded hallway as soon as that individual appeared from one of eight possible entrances. Each participant then tracked the target's path until the individual was no longer visible on the screen (for a demonstration video of task, see https://vimeo.com/395817200).

The visual scene was developed using the Unity 3D game engine version 5.6 (Unity Technologies, San Francisco, CA) and on an Alienware Aurora R6 desktop computer (Alienware Corporation, Miami, FL) with an Intel i5 processor (Intel Corporation, Mountain View, CA), NVidia GTX 1060 graphics card (NVidia Corporation, Santa Clara, CA), and 32 GB of RAM. The 3D human models were created in Adobe Fuse CC and rigged for animation in Adobe Mixamo (Adobe, San Jose, CA), and the 3D object models were created using Blender modeling software (Blender Foundation, Amsterdam, The Netherlands).

**Figure 1.**
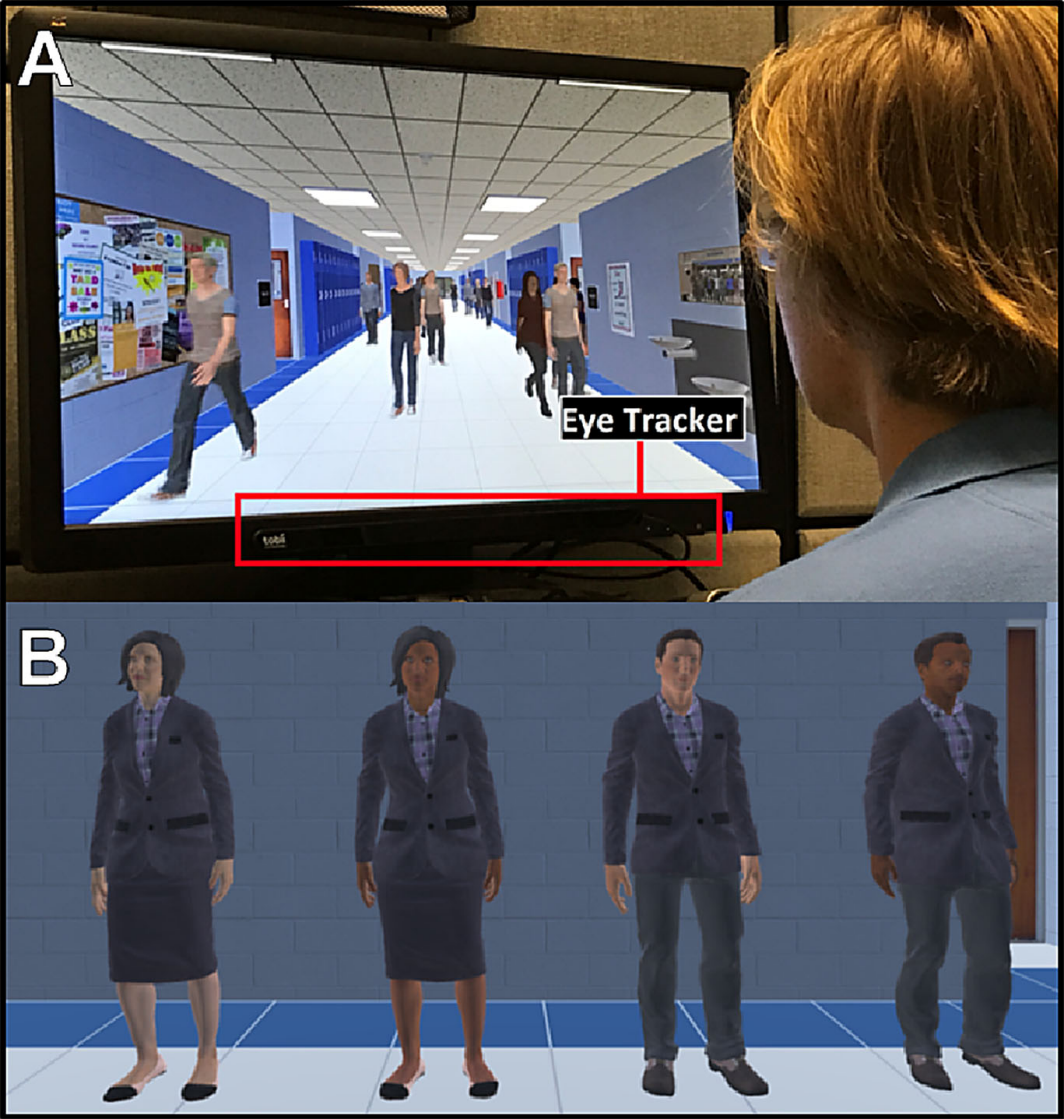
(**A**) Photograph of the apparatus, showing an individual viewing the virtual hallway simulation. (**B**) Visual target selection (the principal of the school) from four possible choices (balanced for gender and race).

Participants were seated comfortably (60 cm away) in front of a 27-in. ViewSonic light-emitting diode, widescreen monitor (1080p, 1920 × 1080 resolution; ViewSonic Corporation, Brea, CA) (Figure 1A). Search patterns (*x*,*y* coordinate positions of gaze on the screen) were captured using the Tobii Eye Tracker 4C (90-Hz sampling rate; Tobii, Danderyd, Sweden). Prior to the first experimental run, eye-tracking calibration was performed for each participant (Tobii Eye Tracking software, version 2.9, calibration protocol) which took less than 1 minute to complete. The process included a seven-point calibration task (screen positions: top–left, top–center, top–right, bottom–left, bottom–center, bottom–right, and center–center) followed by a nine-point post-calibration verification (same seven calibration points plus center–left and center–right). Accuracy was determined by gaze fixation falling within a 2.25° (arc degree) radius around each of the nine points and was further confirmed by inspection prior to commencing data collection.

Participants then viewed and selected their target of choice (i.e., the principal) from four options balanced for gender and race (see Figure 1B). Participants viewed each principal sequentially and independently as they rotated about the *y*-axis plane. Target selection was incorporated in order to enhance the immersive feel of the task and to confirm that the participant was able to correctly identify the principal in isolation before commencing the study. The interval between a target disappearing and reappearing in the hallway from trial to trial varied by 5 to 15 seconds. The duration of the visibility of the target was primarily determined by its starting point and path length; this varied between 5 and 17 seconds for the closest and farthest points, respectively.

**Figure 2.**
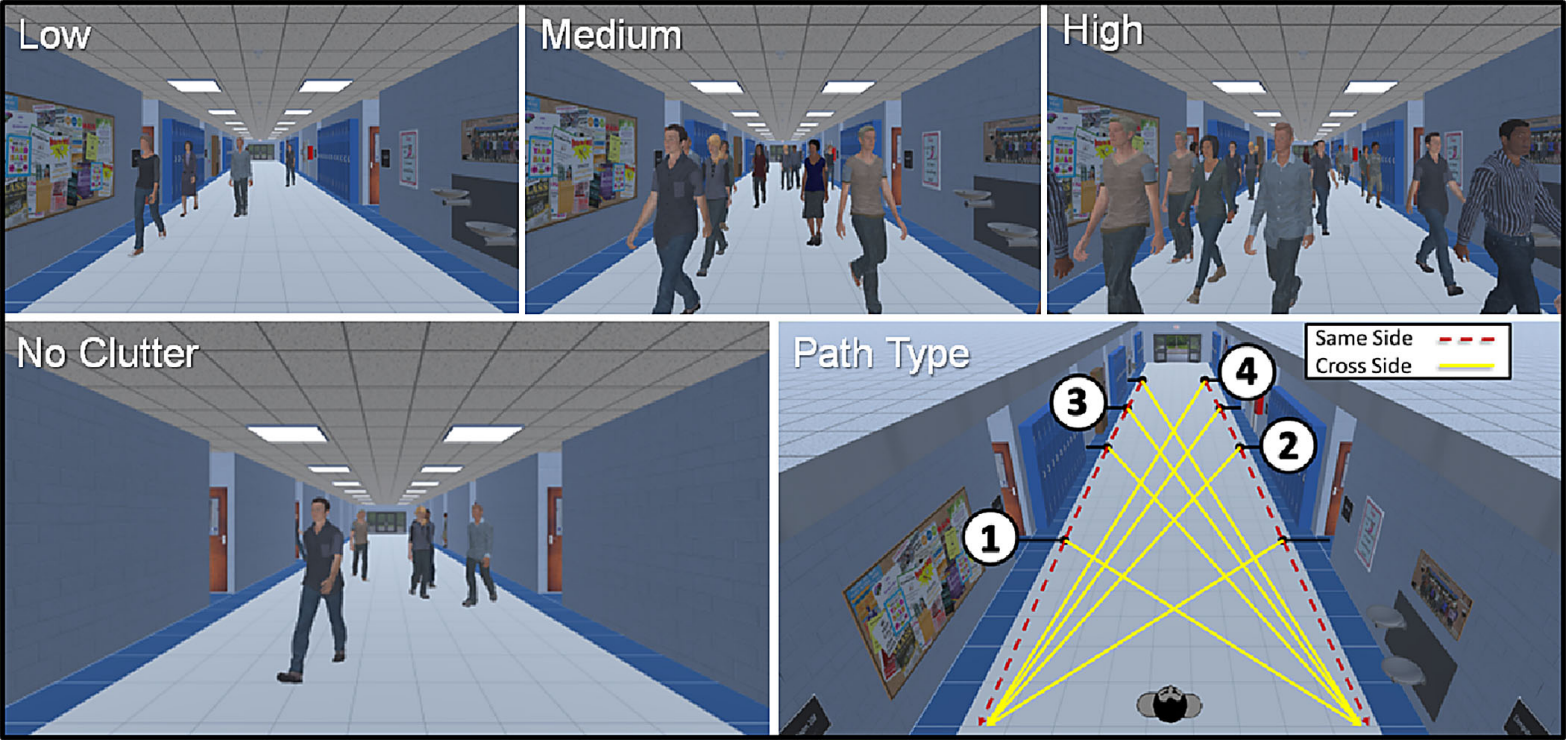
(Top row) Screen shots showing examples of the three levels of crowd density (low, medium, and high). (Bottom left) No-clutter condition in which task-irrelevant features and objects are absent. (Bottom right) Top–down view of the hallway showing the possible target walking paths. Black lines represent the initial start of a target's path into the hallway, dashed red lines are paths taken on the same side as the target entered, and solid yellow lines represent a path crossing in from of the viewer. Distance 1 represents the closest location and distance 4 the farthest, with an equivalent spacing between each of the four distances.

The primary manipulation of interest was crowd density, which was achieved by varying the number of individuals walking in the hallway and ranged from 1 to 20 people. This factor was determined by the number of distractor individuals at a given time and was categorized as low, average of 5 ± 5 people; medium, average of 10 ± 5 people; or high, average of 15 ± 5 people (for examples of each level of crowd density, see Figure 2). Note that there was partial overlap in these ranges, in part because distractors continuously entered and exited the hallway during an experimental run. However, categorization was determined by the sustained average number of distractors present over the course of a specific trial. The second factor of interest was the presence of visual clutter, which was manipulated to investigate the effect of scene complexity on search performance. The visual clutter condition included various objects typically found in a school hallway, such as lockers, water fountains, posters, and pictures. These objects were all absent in the no-clutter condition (for examples of clutter and no-clutter conditions, see Figure 2). Visual clutter was present in 50% and absent in 50% of the trials and interleaved as part of a pseudorandom presentation order.

As part of the stimulus design, the target path trajectory was also varied. Specifically, the principal's walking path could originate from one of eight possible locations in the hallway, based on four possible starting distances and entering from either the right or left of the observer. The target would continue walking, either crossing in front of or remaining on the same side as the viewer (for path type options, see Figure 2).

Participants completed three runs of the experiment with a brief rest period in between. Each run lasted approximately 3.5 minutes. Within a run, participants experienced an equal amount of trials for the two primary factors of interest (i.e., crowd density and visual clutter) and their respective conditions. Over the course of three runs, these factors were pseudorandomized and balanced in terms of presentation. The paths the target walked were not fully randomized but instead were constrained in order to ensure an equal sampling of starting positions (close vs. far points), path type (crossing vs. same side), and side of hallway (starting from the left vs. right side). As an example, a participant would have the same number of trials where the target crossed the screen for the low, medium, and high crowd densities, but not from every starting point or side. Thus, for each level of crowd density, participants experienced an equal number of trials for left/right starting points and door distance (but not every possible combination was covered for each level of crowd density). This was done to allow for a simpler factorial design while ensuring that the target path variables did not confound with the two primary manipulations of interest (crowd density and visual clutter).

### Data capture, analysis, and outcome measures

Eye-tracking data characterizing visual search performance and responses were captured as participants initially located (via a saccade) and followed (via smooth pursuit) the target on the screen. The captured data represented on-task gaze activity, defined as periods when the target principal was visible on the screen. After data collection, all gaze data were centered point by point relative to the location of the target. Centering of the data was done to provide a common point of comparison so that all collected search data could be referenced across trials and conditions. Given that the target was moving, gaze data were updated on a frame-by-frame basis to preserve their respective location throughout the pursuit of the target. This allowed for the generation of heat maps to visualize the density of gaze point distribution around the target across trials and conditions of interest. From these centered data, several measures were used to objectively quantify performance.

#### Heat maps

At a first level, heat maps were generated to visualize the extent and distribution patterns of gaze data (Bennett et al., 2018a, 2018b; Gibaldi, Vanegas, Bex, & Maiello, 2017); for further discussion of this approach, see Blascheck, Kurzhals, Raschke, Burch, Weiskopf, & Ertl, 2014; Blignaut, 2010; Špakov & Miniotas, 2007). The process for generating heat maps used here was to smooth and aggregate centered gaze data over time using histogram regional binning and Gaussian filtering (Gibaldi et al., 2017). The varying colors represent differing levels of gaze data density across spatial regions of the screen, with each color corresponding to a probability of fixation at that location. Specifically, yellow indicates that a participant spent a large portion of time looking at a particular area, whereas blue indicates areas where a participant spent less time (with approximately a 9:1 ratio of point density between yellow and blue) (Bennett et al., 2018a; Bennett, Bailin, Gottlieb, Bauer, Bex, & Merabet, 2018b). It is also important to note that the resultant image is a representation of overall task performance based on the centering of data and not the actual position of the target for a given trial. Areas without any color heat map correspond to regions where there were insufficient points to meet a minimum data capture threshold and thus are not indicative of a complete lack of gaze exploration.

#### Ellipse of best fit

The primary outcome of interest was an ellipse area representing a 95% confidence interval for the captured eye-tracking data. This was expressed as a percentage of the screen area and represents a measure of visual search precision. Specifically, the ellipse area extends around the perceived centroid location of the target and indicates how well a person searched and continued pursuit within the area where they perceived the target to be (precision around spatial centroid). Thus, the confidence ellipse area measure includes both components of the search and pursuit processes.

#### Gaze error

Gaze error (expressed in arc degrees) corresponds to the distance between the center of the target and the participant's gaze position (Kooiker, Pel, van der Steen-Kant, & van der Steen, 2016; Pel, Manders, & van der Steen, 2010). This was computed based on the sampling rate of the eye tracker (90 Hz) and serves as a continuous measure of overall locating and pursuit accuracy of the target stimulus.

#### Reaction time

We analyzed reaction time as a measure of cognitive processing and visual search ability. Reaction time was defined as the first moment the participant's gaze arrived within the outer contour of the target and pursued the target on the screen for a given trial (Bennett et al., 2018a; Bennett et al., 2018b). Pursuit was defined as gaze that remained within the outer contour of the target for a minimum of 400 ms. Visibility of the target was determined based on the viewer's perspective. In the case where the target entered the hallway and was partially occluded from view, the first moment they became visible to the viewer would mark the start of the reaction time calculation.

#### Classification of eye movements, pursuit/saccade ratio

Each individual data point was compared to the previous data point (sequentially in time) to determine the speed of eye movement (expressed in arc degrees per second). All collected gaze data were then classified as either saccade or pursuit eye movements. Saccades were defined as eye movements that exceeded 25 arc degrees per second, and pursuits were defined as eye movements less than 25 arc degrees per second, following criteria outlined from previous related studies (Leigh & Zee, 2015; Shibata, Tabata, Schaal, & Kawato, 2005). Note that microsaccades during fixation on static objects were classified as pursuit following these same criteria. From these dichotomized data, a ratio was calculated by dividing the number of pursuit points by the number of saccade points within any given trial. A pursuit/saccade ratio greater than 1 is indicative of more time spent gazing at individual objects rather than switching gaze between objects and is consistent with more target identification and tracking and less time spent searching.

#### Success rate, off-screen count, and reliability

The success rate was defined by whether the participant was able to locate and maintain pursuit of the target. A trial was considered unsuccessful if a participant failed to locate and establish pursuit for 400 ms (as specified for reaction time above) prior to the target leaving the screen (end of trial). A percentage was then calculated based on the condition level of a given trial, and an average for the total number of trials was extracted.

Because the position of the participant's gaze on the screen was constantly recorded, we also determined how often and how long they looked at and away from the screen on a given trial. The off-screen counts represent the number of gaze points that fell outside of the bounds of the screen. Given the known frequency at which the gaze data were being logged (90 Hz), each off-screen data point can be expressed as specific amount of time. This off-screen count served as an index of testing compliance and adherence to task instructions, as well as overall level of engagement.

Finally, overall test–retest reliability was analyzed using a Bland–Altman test of repeatability (Bland & Altman, 1986; Bland & Altman, 2010) based on all of the outcome measures of interest. This was determined by directly comparing an individual's performance on the first run to their second run of the task followed by comparing an individual's performance on the first run to their third run of the task. Test–retest was done to evaluate the spread of variance and result consistency, as well as to explore any potential cumulative effects over time.

#### Statistical analysis of captured data

A univariate analysis of variance (ANOVA) was conducted for each of the four primary measures (confidence ellipse, gaze error, reaction time, and pursuit/saccade ratio). Each ANOVA included the two primary factors of crowd density and presence of visual clutter. Mauchly's test was used to confirm that the assumption of sphericity was not violated for any of the ANOVAs. *Post* *hoc* Bonferroni corrected *t*-test comparisons were conducted for significant main effects. A subanalysis was also conducted that specifically focused on the target path variables. A univariate ANOVA was also run for each of the primary measures and included starting distance (close vs. far points) and path type (crossing vs. same side). Statistical analyses were performed using SPSS Statistics (IBM, Armonk, NY). Statistical significance was set at *p* < 0.05.

## Results and data interpretation

### Visualization of visual search data

**Figure 3.**
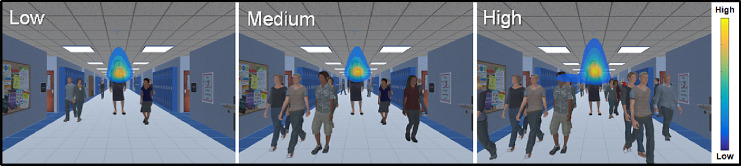
Heat maps illustrating the extent of eye movement search patterns overlaid on top of representative examples of low, medium, and high crowd density levels of visual distractors. The color scheme of the heat maps represents differing levels of gaze data density across spatial regions of the screen space (yellow indicates more time looking in an area, and blue indicates less time). There is consistent clustering of eye movements across the levels of distractor density, reflecting a tight flame shape around the target as they locate and pursue. Note further dispersion of gaze data from the target as crowd density increases.

Heat maps showing the overall distribution of the group average on the three levels of crowd density are presented in Figure 3. Note the general flame pattern (associated with sustained pursuit of the target along its trajectory and tight clustering of search area around the upper region of the target (i.e., the head and chest area of the principal). Qualitatively, there was also a trend for increased visual search area from low to medium to high crowd densities (with the largest distribution of search area seen at the high crowd density level). This qualitative observation suggests that overall gaze area increased with increasing crowd density, and participants focused on the upper features of the human target (e.g., head and chest) for the purposes of identification and tracking. This observation is consistent with previous research demonstrating selective attention to specific areas (such as faces) in scenes containing people (Cerf, Harel, Einhäuser, & Koch, 2008; Crouzet, Kirchner, & Thorpe, 2010).

### Effect of crowd density

#### Confidence ellipse area

**Figure 4.**
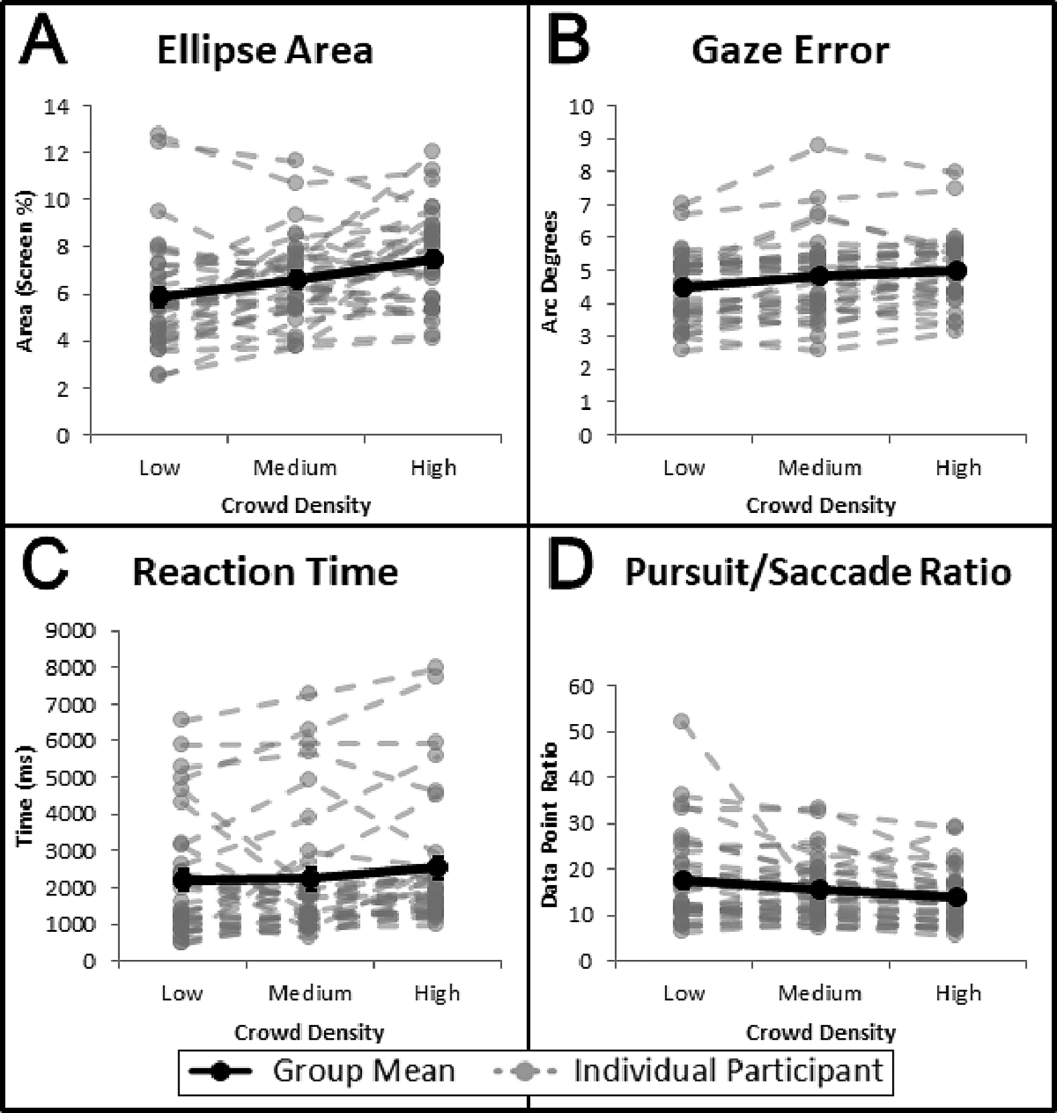
Group mean (black solid lines) and individual mean (gray dashed lines) data are shown for the four performance outcomes of interest and across the three levels of crowd density. (A) Ellipse area, (B) gaze error, (C) reaction time, and (D) pursuit/saccade ratio. Standard errors are shown for the group means (some not visible).

Figure 4A shows the overall mean ellipse area (black data) and individual results for each participant (gray data) as a function of crowd density, with group means of 5.85% (low), 6.63% (medium), and 7.47% (high). In general, there was a trend for increasing ellipse area with greater crowd density (an increase in area from low to high density of 27.7%). An ANOVA showed a significant main effect of crowd density, *F*(2, 34) = 15.167, *p* < 0.001, η_p_^2^ = 0.031. There was no significant interaction effect between the primary manipulations (crowd density and clutter presence) for ellipse area, *F*(2, 34) = 0.268, *p* = 0.765, η_p_^2^ = 0.001. Further details on the main effect of clutter presence are presented in below. Bonferroni-corrected *post hoc* pairwise comparisons revealed significant differences between each pairing of low versus medium (*p* = 0.025), low versus high (*p* < 0.001), and medium versus high (*p* = 0.014) crowd densities. The trend for increasing ellipse area with increasing crowd density is consistent with qualitative observations obtained from the heat map distribution patterns and suggests that greater crowd density (i.e., increased number of distractors) is associated with decreased search precision.

#### Gaze error

Figure 4B shows the overall mean for gaze error (black data) and individual results (gray data) as a function of crowd density, with group means of 4.48° (low), 4.83° (medium), and 5.03° (high). In general, there was a trend for increasing gaze error with greater crowd density (an increase from low to high of 12.3%), although this trend was not as pronounced for ellipse area (27.7%) in terms of magnitude. ANOVA results for gaze error revealed a significant effect of crowd density, *F*(2, 34) = 6.069, *p* = 0.002, η_p_^2^ = 0.013. No significant interaction effect was found between the primary manipulations (crowd density and clutter presence) for gaze error, *F*(2, 34) = 1.360, *p* = 0.257, η_p_^2^ = 0.003. Further details on the main effect of clutter presence are presented below. Bonferroni-corrected *post hoc* pairwise comparisons revealed significant differences only between the low versus high conditions (*p* = 0.001) but not for the low versus medium (*p* = 0.07) or medium versus high (*p* = 0.64) conditions. This trend suggests that accuracy of eye movements decreased (as indexed by increasing gaze error) with increasing crowd density. Although this trend was also evident for the ellipse area outcome, the magnitude of effect was not as pronounced.

#### Reaction time

Figure 4C shows the overall mean (black data points) and individual participant (gray data points) reaction time as a function of crowd density, with group means of 2212 ms (low), 2239 ms (medium), and 2545 ms (high). Similar to confidence ellipse area and gaze error, there was a trend for increased reaction time with increasing crowd density (an increase from low to high of 15.1%); however, an ANOVA revealed that this trend was not statistically significant, *F*(2, 34) = 1.519, *p* = 0.220, η_p_^2^ = 0.003. No significant interaction effect was seen in the primary manipulations (crowd density and clutter presence) for reaction time, *F*(2, 34) = 0.184, *p* = 0.832, η_p_^2^ < 0.001. Further details on the main effect of clutter presence are presented below. This may be due to the comparatively large intraindividual variability observed, a byproduct of having multiple starting distances for the target. Further examination of the effect of target starting distance and a potential explanation for the lack of an observed statistically significant effect can be found below as a separate subanalysis.

#### Pursuit/saccade ratio

Figure 4D shows the overall average (black data points) and individual (gray data points) pursuit/saccade ratios as a function of crowd density, with group means of 17.55 (low), 15.66 (medium), and 13.82 (high). There was a trend for decreasing pursuit/saccade ratio as a function of increasing crowd density (a decrease from low to high of 21.3%). These results show that, as crowd density increased, more time was spent searching for the target rather than actively pursuing it. An ANOVA confirmed a significant effect of crowd density, *F*(2, 34) = 6.231, *p* = 0.002, η_p_^2^ = 0.013. No significant interaction effect was found between the primary manipulations (crowd density and clutter presence) for the pursuit/saccade ratio, *F*(2, 34) = 0.137, *p* = 0.872, η_p_^2^ < 0.001. Further details on the main effect of clutter presence are presented below. *Post* *hoc* comparisons revealed that there were significant differences between the low and high conditions (*p* = 0.001) but not between the low and medium (*p* = 0.215) or medium and high (*p* = 0.232) conditions. This outcome once again supports the notion that increased crowd density is associated with greater search difficulty, and participants spent more time searching (i.e., saccades) rather than pursuing the target at higher crowd densities.

### Effect of presence of clutter

The mean values for each outcome measure are summarized in Table 1. In general, the results suggest that the presence of clutter across all outcomes of interest did not have a significant effect. The respective ANOVA outcomes for ellipse area, *F*(1, 34) = 0.004, *p* = 0.947, η_p_^2^ < 0.001; gaze error, *F*(1,34) = 0.047, *p* = 0.829, η_p_^2^ < 0.001; reaction time, *F*(1, 34) = 3.177, *p* = 0.075, η_p_^2^ = 0.003; and pursuit/saccade ratio, *F*(1, 34) = 0.309, *p* = 0.578, η_p_^2^ < 0.001, confirmed this result. Although there was a trend for increasing reaction time with the presence of clutter, this did not reach statistical significance.

**Table 1.** Mean group values for the four primary outcome measures broken down by trials with and without clutter.

Measure	No clutter	Clutter
Ellipse area (screen %)	6.66	6.65
Gaze error (arc degrees)	4.79	4.77
Reaction time (ms)	2171	2486
Pursuit/saccade ratio	15.92	15.43

**Figure 5.**
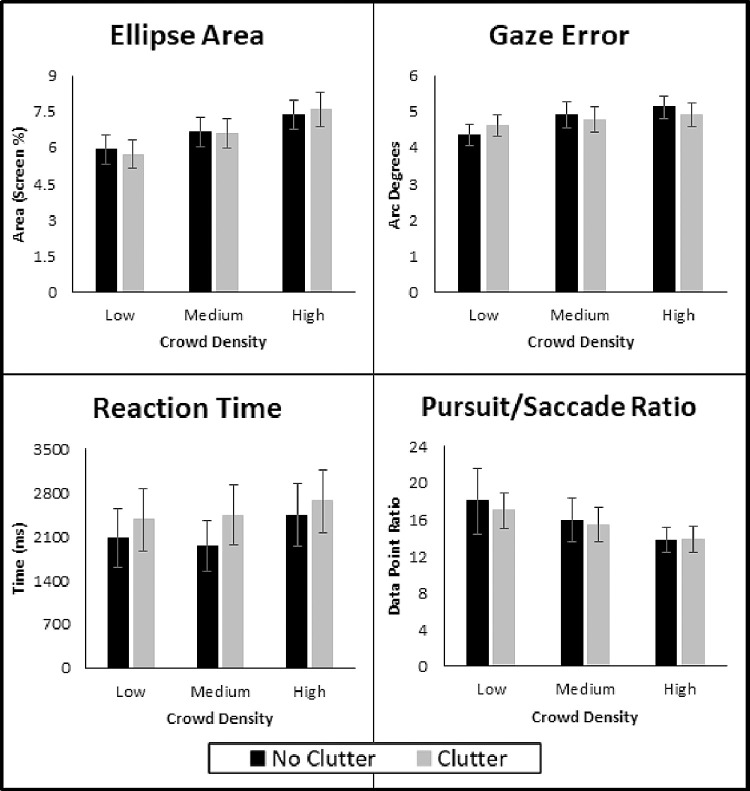
Group mean data are shown for the four performance outcomes of interest and are displayed across the three levels of crowd density and two levels of clutter presence. (**A**) Ellipse area, (**B**) gaze error, (**C**) reaction time, and (**D**) pursuit/saccade ratio. Standard errors are shown for group means.

As mentioned previously, no significant interaction effects were observed between crowd density and clutter presence. Figure 4 focuses on crowd density and displays the individual participant data, and Figure 5 breaks down the group data of each measure by the respective levels of each factor.

### Success rates, off-screen counts, and test–retest reliability

Overall, there were minimal differences in the success rates of finding the target for the primary manipulations. In general, success rates were high and ranged from 94% to 98% (i.e., 94.6% for low, 97.5% for medium, and 97.8% for high crowd densities). There were no statistically significant differences in success rates across the manipulations of interest—crowd density, *F*(2, 34) = 2.630, *p* = 0.076, η_p_^2^ = 0.006; visual clutter presence, *F*(1, 34) = 1.061, *p* = 0.300, η_p_^2^ = 0.001—or an interaction of these factors, *F*(2, 34) = 0.283, *p* = 0.754, η_p_^2^ = 0.001. Of note, we observed a trend for lower success rates with the lowest complexity condition levels of crowd density and presence of visual clutter (Table 2). For clutter, this result is consistent with classic visual search paradigms in which set size has little effect for targets that can easily be segmented (Nakayama & Silverman, 1986; Teichner & Krebs, 1974). The absence of an effect of crowd size is not consistent with this explanation, as the trajectories of the distractors are similar to that of the target, which has been shown to impair search performance (Horowitz et al., 2007). However, others have shown that conjunction searches for form and motion may not manifest a set size effect (McLeod, Driver, & Crisp, 1988), and our results are consistent with this observation. Finally, this observation could be potentially related to a lack of engagement when little activity is occurring on screen.

**Table 2.** Mean group values for the off-screen count (percent of points per trial) and success rate measures broken down by crowd density (low, medium, and high conditions) and trials with or without clutter.

Measure	Low	Medium	High	No clutter	Clutter
Off-screen count (%)	0.38	0.29	0.28	0.38	0.26
Success rate (%)	94.60	97.46	97.78	95.89	97.31

In an attempt to quantify potential lapses in attention, we examined the proportion of gaze data points falling outside of the screen. Table 2 summarizes the off-screen counts across all of the manipulations of interest. Considering that trials typically average around 1000 data points, off-screen points represent only a tiny fraction of total points per trial, ranging from 0% to 1%; however, they are still meaningful in picking up changes in eye movements away from the task and adherence to task instructions. As an example, a reduction in off-screen points during the high distractor trials was observed, and this may indicate that participants were more engaged and less likely to look away from the screen when more activity was occurring. This is consistent with an observed trend of lower success rates at lower levels of task difficulty (although this trend did not reach statistical significance).

Test–retest agreement for the visual search task was examined using a Bland–Altman analysis between individual runs within each participant and for all outcomes of interest. This was performed in order to investigate consistency across runs and to demonstrate congruency between outcome measures. The Bland–Altman test revealed a low coefficient of variation for confidence ellipse (run 1 vs. run 2 = 0.24; run 1 vs. run 3 = 0.25), gaze error (run 1 vs. run 2 = 0.13; run 1 vs. run 3 = 0.18), reaction time (run 1 vs. run 2 = 0.53; run 1 vs. run 3 = 0.52), and pursuit/saccade ratio (run 1 vs. run 2 = 0.22; run 1 vs. run 3 = 0.25). Coefficients of repeatability were also determined for confidence ellipse (run 1 vs. run 2 = 0.03; run 1 vs. run 3 = 0.04), gaze error (run 1 vs. run 2 = 1.20; run 1 vs. run 3 = 1.68), reaction time (run 1 vs. run 2 = 2.41; run 1 vs. run 3 = 2.37), and pursuit/saccade ratio (run 1 vs. run 2 = 6.98; run 1 vs. run 3 = 7.67). Finally, no significant bias was found for any of the measures (all *p* > 0.29). These results indicate that the performance of each participant remained consistent and balanced across runs.

### Effect of target path

We classified paths as either crossing (the target crossed in front of the viewer) or same side (the target remained on one side). Starting positions for the target also occurred at four different distances. The mean values for each outcome measure are summarized in Table 3. The ANOVA revealed statistical significance of path type for gaze error, *F*(1, 34) = 4.112, *p* = 0.043, η_p_^2^ = 0.004, and reaction time, *F*(1, 34) = 4.657, *p* = 0.031, η_p_^2^ = 0.005, but not for ellipse area, *F*(1, 34) = 0.087, *p* = 0.768, η_p_^2^ < 0.001, or pursuit/saccade ratio, *F*(1, 34) = 0.747, *p* = 0.388, η_p_^2^ = 0.001. Of note, the largest increase observed was for reaction time, which was 16.34% higher for same-side paths than for crossing paths. This result could be explained by the fact that targets crossing in front of the observer are easier to detect, and targets on the same side require greater attention to the periphery. The ANOVA also showed statistical significance for target starting distance and for all measures: ellipse area, *F*(3, 34) = 4.805, *p* = 0.003, η_p_^2^ = 0.015; gaze error, *F*(3, 34) = 238.725, *p* < 0.001, η_p_^2^ = 0.435; reaction time, *F*(3, 34) = 106.972, *p* < 0.001, η_p_^2^ = 0.263; and pursuit/saccade ratio, *F*(3, 34) = 13.922, *p* < 0.001, η_p_^2^ = 0.043. However, the effect was mixed between positive and negative for the various measures.

**Table 3.** Mean group values for the four primary outcome measures broken down by path type (trials where the target's path stayed on the same side or crossed in front of the viewer) and for the four starting distances (distance 1 being closest to the viewer and distance 4 the farthest).

Measure	Cross path	Same path	Distance 1	Distance 2	Distance 3	Distance 4
Ellipse area (screen %)	6.60	6.17	7.29	6.82	6.13	6.27
Gaze error (arc degrees)	4.69	4.88	6.72	4.79	3.87	3.47
Reaction time (ms)	2161	2514	758	1544	2578	4588
Pursuit/saccade ratio	16.03	15.30	11.56	15.38	18.22	18.10

**Figure 6.**
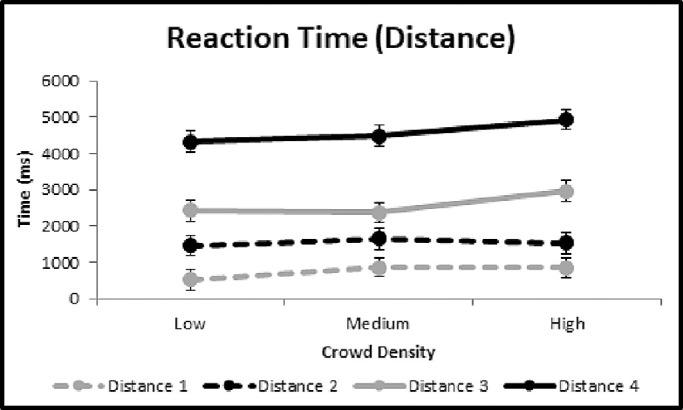
Group mean reaction time data are shown across the three levels of crowd density and broken down among the four distances at which the target can begin the walking path in the hallway. Standard errors are shown (some not visible).

In particular, reaction time showed the largest effect of starting distance with a sixfold difference between the closest and farthest distances. As mentioned previously, this may explain the lack of a significant effect observed with respect to crowd density for the reaction time measure as it was superseded by the effect of starting distance. Further illustrations of the relationship between reaction time and the four starting distances across each level of crowd density are shown in Figure 6.

## Conclusions

The present work investigates dynamic visual search performance using a VR-based task combined with eye tracking in a sample of participants with neurotypical development. Our approach incorporates control of task features that are easy to understand and implement. Furthermore, their effects on search performance can be characterized through an analysis of ocular motor behavior in a naturalistic dynamic visual scene. The high behavioral relevance of the task may also find utility in assessing functional visual performance in other clinical populations of interest (Parsons, 2015; Parsons et al., 2017).

### Summary of effects and manipulations

Overall, our results showed a pattern of worsening performance with increasing task difficulty. The effect of crowd density was statistically significant for all outcome measures except reaction time. In general, the greatest differences were observed between the low and high condition levels. In contrast, the presence of visual clutter was not found to significantly affect performance for any of the measures. Finally, by assessing success rates and off-screen counts, we observed an overall high level of performance on this task.

As previously mentioned, the present work serves as a characterization of baseline performance for this VR-based visual search task in a sample of controls with neurotypical development. In this context, the overall high level of performance on each measure is not that surprising. However, the characterization of these trends and metrics may be important for future applications in other clinical populations of interest, such as pediatric and individuals with visual and/or cognitive impairments. This is particularly true with respect to characterizing performance with respect to varying task demand beyond what is revealed using standard ophthalmic assessments such as visual acuity and visual perimetry. Finally, the reliability and repeatability of the task (as confirmed by the Bland–Altman analyses) are also important factors for establishing the validity of this testing approach.

### Strengths of visual stimulus and study design

The present work offers strengths related to the overall design and development of the visual stimulus and task, as well as a range of behavioral outcomes and statistical measures. The stimuli and protocol were designed in a manner to provide a realistic simulation to investigate the effect of manipulating factors of interest (specifically, crowd density and presence of visual clutter). In contrast to previous studies (e.g., using point light walkers to simulate biological motion), we present realistic 3D human models while allowing for the control of multiple feature conjunctions. The objective measures were chosen and designed to be straightforward and reproducible, and they individually add to a full characterization of task performance, including comprehensive measures focused on visual search indexing accuracy (gaze error), precision (confidence ellipses), cognitive efficiency (reaction time), ocular movement type (pursuit/saccade ratio), repeatability (test–retest), task compliance (off-screen counts), and task completion (success rate).

### Potential use of virtual reality with other populations

An important feature of the current work is the application of a naturalistic and behaviorally relevant task combining VR and eye tracking to assess search performance in a dynamic and complex naturalistic scene. Traditional task design tends to focus on stimulus and experimental control, thereby forgoing realism; however, the work presented here demonstrates an integration of both aspects. Furthermore, the current task does not rely on physical (e.g., button press) or verbal responses. As long as the participant is able to understand the task, tracking eye movements are the only requirement for task performance monitoring. This fact increases the accessibility of the VR task for populations where physical or cognitive disabilities may be a significant factor. Given also the straightforward, realistic, and engaging nature of the task, this also opens up possibilities for assessing visual search performance in a variety of clinical populations of interest (Parsons, 2015; Parsons et al., 2017).
